# Preclinical and post-treatment changes in the HCC-associated serum proteome

**DOI:** 10.1038/sj.bjc.6603429

**Published:** 2006-10-24

**Authors:** D G Ward, Y Cheng, G N'Kontchou, T T Thar, N Barget, W Wei, A Martin, M Beaugrand, P J Johnson

**Affiliations:** 1 Cancer Research UK Institute for Cancer Studies, University of Birmingham, Edgbaston, Birmingham, B15 2TT, UK; 2 APHP Hospital Jean Verdier, Bondy and Université Paris 13, Paris, France

**Keywords:** hepatocellular carcinoma, serum, proteome, SELDI

## Abstract

SELDI-based proteomic profiling of body fluids is currently in widespread use for cancer biomarker discovery. We have successfully used this technology for the diagnosis of hepatocellular carcinoma (HCC) in hepatitis C patients and now report its application to serial serum samples from 37 hepatitis C patients before development of HCC, with HCC and following radiofrequency ablation of the tumour. As with alpha-fetoprotein, an accepted biomarker for HCC, we hypothesised that HCC-associated proteomic features would ‘return to normal’ following successful treatment and the primary aim of our study was to test this hypothesis. Several SELDI peaks that changed significantly during HCC development were detected but they did not reverse following treatment. These data may be interpreted to suggest that the characteristic SELDI profile is not linearly related to tumour burden but may result from the progression of underlying liver disease or from the emergence of precancerous lesions. *β*2-Microglobulin, a protein previously reported to be markedly elevated in patients with HCV related HCC, was also the most significantly HCC associated proteomic feature (*m*/*z* 11720) in this study.

Early diagnosis remains the key to effective therapy of HCC. When diagnosed below the size of 3 cm several surgical or ablative options are available which are potentially curative (DeMatteo *et al*, 1999; Belghiti, 2005; Jansen *et al*, 2005). However, symptoms seldom develop in patients with these small tumours with the result that relatively few patients are suitable for such treatment options. There is, therefore, considerable interest in presymptomatic detection by surveillance of high-risk groups (Liaw *et al*, 1986; Colombo *et al*, 1991; Chalasani *et al*, 1999; McMahon *et al*, 2000; Mok *et al*, 2005). Although there is no conclusive evidence (in terms of reduction of disease-specific mortality) that surveillance is effective, it is nonetheless widely practiced (Chalasani *et al*, 1999) and there is no doubt that such programmes do result in the detection of small tumours (Chalasani *et al*, 1999; Mok *et al*, 2005). The two currently applied approaches to early detection are serial estimation of serum alpha fetoprotein (AFP, a serological tumour marker for HCC) and ultrasound examination. However, the former is so insensitive that many have questioned its usefulness, and indeed a recent international consensus statement has suggested that AFP should no longer be used for this purpose (Sherman, 2001; Trevisani *et al*, 2001; Anon, 2005).

Although a non-invasive test would clearly be useful, at present there does not seem to be any other serological marker that could improve on AFP and alternative strategies need to be investigated. One such approach is proteomic profiling (Adam *et al*, 2002; Li *et al*, 2002; Kozak *et al*, 2003; Poon *et al*, 2003; Chen *et al*, 2004; Paradis *et al*, 2005; Schwegler *et al*, 2005; Yang *et al*, 2005; Ward *et al*, 2006a, 2006b and 2006c). We and other groups have suggested that patients with HCC may express a characteristic protein ‘signature’ even in the case of small tumours (Poon *et al*, 2003; Paradis *et al*, 2005; Schwegler *et al*, 2005; Ward *et al*, 2006a, 2006b and 2006c). These signatures are customarily detected using Surface-Enhanced Laser Desorption/Ionisation (SELDI) technology, which permits the mass spectrum of a specific subset of serum proteins to be compared between cancer and non-cancer patients using various pattern recognition programmes. While there has been considerable controversy about the robustness of this approach, particularly with regard to its reproducibility and the techniques used to validate results (Diamandis, 2004; Petricoin *et al*, 2004; Ransohoff, 2004), a large body of evidence does suggest that such signatures are detectable (Adam *et al*, 2002; Li *et al*, 2002; Kozak *et al*, 2003; Poon *et al*, 2003; Chen *et al*, 2004; Paradis *et al*, 2005; Schwegler *et al*, 2005; Yang *et al*, 2005; Ward *et al*, 2006a, 2006b and 2006c), even if clinical utility remains to be convincingly shown.

We have previously assessed the validity of this approach by testing the signature on a blinded ‘validation data set’, held externally to the Unit undertaking the SELDI analysis (Ward *et al*, 2006b), A second validation method is to determine the changes consequent upon effective treatment, and changes that occur prior to clinical presentation. In the case of AFP, the classical biomarker for HCC, such changes are well documented. Prior to clinical presentation there is a steady exponential rise (with a typical doubling time of around 30 days) in AFP levels. After effective treatment levels fall immediately with a half life of around 5 days (Johnson and Williams, 1980).

In this study, using a cohort of patients in whom we have previously defined and validated characteristic proteomic signatures (Ward *et al*, 2006b), we now examine the changes that occur before the clinical presentation of HCC and after effective treatment, testing the hypothesis that the ‘signature’ will behave like a classical tumour marker, and return towards normal.

## PATIENT AND METHODS

### Sample collection

Serum samples were collected between May 1994 and January 2005 at Jean Verdier Hospital, Bondy, France. Sample collection was officially registered and all patients gave informed consent. Sera were stored at −80°C. All patients were hepatitis C positive and HCC was diagnosed histologically or non-invasively, according to the EASL Barcelona Conference criteria (Bruix *et al*, 2001). Samples were transported on dry ice to the University of Birmingham, UK, for analysis in November 2005, defrosted and multiple 20 *μ*l aliquots taken and stored at −80°C pending SELDI analysis. Quality control (QC) samples were prepared by mixing equal volumes of serum from 27 healthy individuals and stored as multiple aliquots at −80°C.

### Study design

We studied 91 samples from 37 patients with HCC (age range 47–83, median age 63 years, 22 male patients and 15 female patients). Thirty-eight samples were taken on average 38 months (range 8–123 months) before the diagnosis of HCC, 35 samples taken once HCC had become clinically evident and 18 sera taken on average 15 months (range 1–52 months) after treatment. Tumours ranged from 10 to 45 mm in diameter. In all cases, treatment was on the basis of radiofrequency ablation. All of the post-treatment samples were from patients considered to have achieved complete local control.

Independent duplicate SELDI spectra were collected for all serum samples using Cu^2+^ loaded IMAC30 proteinchip arrays. Samples were processed using 96-well bioprocessors and the sample order on each bioprocessor was randomised with duplicates on separate bioprocessors.

### SELDI procedure

Sera were analysed using Cu^2+^ loaded IMAC30 proteinchip arrays as described previously (Ward *et al*, 2006b). Briefly, the proteinchip arrays were pretreated with CuSO_4_ followed by a water rinse and equilibration with binding buffer (500 mM NaCl, 100 mM NaH_2_PO_4_/NaOH (pH 7.0)). Serum samples were diluted 5-fold with 9 M urea, 2% CHAPS, 50 mM Tris/HCl (pH 9.0) followed by 10-fold dilution in binding buffer. These 50-fold diluted samples were loaded on proteinchip arrays (100 *μ*l per spot) using a 96-well bioprocessor and incubated at room temperature for 1 h. Following washing with binding buffer and a water rinse 2 × 1 *μ*l of 50% saturated sinapinic acid in 50% acetonitrile/0.5% trifluoroacetic acid was added per spot. The proteinchip arrays were analysed in a PBSIIc SELDI-TOF equipped with an autoloader (Ciphergen). Spectra were collected over mass to charge (*m*/*z*) ratios of 0–20 000 and 0–200 000 (488 laser shots) using laser intensities of 165 and 210, respectively. Spectra were externally calibrated in the *m*/*z* 0–20 000 range using all-in-one peptide standard (Ciphergen) with added cytochrome *C* and myoglobin (Sigma-Aldrich, Poole, Dorset, UK). The *m*/*z* 0–200 000 range was calibrated using chymotrypsinogen, bovine serum albumin and phosphorylase b (Sigma). Spectra were normalised using the total ion current from *m*/*z* 2000–20 000 and 20 000–200 000. Peaks were selected and clustered using Biomarker Wizard software (Ciphergen) with the signal to noise ratio >5 for the first pass and >2 for the second, a cluster mass window of 0.2%, and a requirement for peaks to be present in >20% of the spectra. These settings resulted in 125 peaks being selected. The peak intensities from the duplicate spectra from each sample were averaged and the resulting peak intensities compared between pre-HCC and HCC samples and between HCC and post-treatment samples. To give an estimate of experimental variability the intensities of 82 peaks were compared across 10 replicates of a QC sample assayed randomly through the study. The average coefficient of variation of peak intensity was 21%.

### Peak identification

The protein underlying the most significantly HCC associated proteomic feature at *m*/*z* 11720 was purified, trypsinised and identified by LC-MS/MS. SELDI was used to monitor the purification. A pool of sera with high levels of the *m*/*z* 11 720 proteomic feature was diluted five-fold with 9 M urea, 2% CHAPS, 50 mM Tris/HCl (pH 9.0). This denatured serum was then applied to Q Ceramic Hyper D anion exchange resin (Pall) and proteins eluted with washes of decreasing pH. The *m*/*z* 11720 peak eluted at pH 7 and was subsequently applied to a C18 reverse phase column (Vydac) in 0.1% TFA. Proteins were eluted with an acetonitrile gradient, and the fractions containing the *m*/*z* 11720 peak (54–60% acetonitrile) were lyophilised and run on a 12% nonreducing gel using MES buffer (Invitrogen Ltd., Paisley, Scotland). A band with ∼12 kDa mobility was excised from the gel, digested with trypsin and the resulting peptides analysed on a Thermofinnigan Deca XP ion trap mass spectrometer as described previously (Ward *et al*, 2006b, 2006c). An ‘immunoSELDI’ approach was used to confirm that the identified protein corresponded to the SELDI peak at *m*/*z* 11720. A rabbit polyclonal antibody (Sigma: M8523) raised against human *β*2-microglobulin was bound to Protein-G beads and added to serum diluted 10-fold in 150 mM NaCl, 20 mM MOPS (pH7.4), 0.1% *n*-octylglucoside. The SELDI spectrum of the ‘depleted’ serum was compared with that of serum incubated with Protein-G beads in the absence of antibody. Additionally, the beads were washed with buffer and then 50% acetonitrile/0.5% trifluoroacetic acid. The eluted proteins were also analysed by SELDI.

### Estimation of serum AFP concentrations

AFP was measured in all samples in one batch using an AFP ELISA kit (DRG International) according to the manufacturer's instructions.

## RESULTS

### Comparison of serum profiles before and after development of clinically detectable HCC

**Figure 1:**
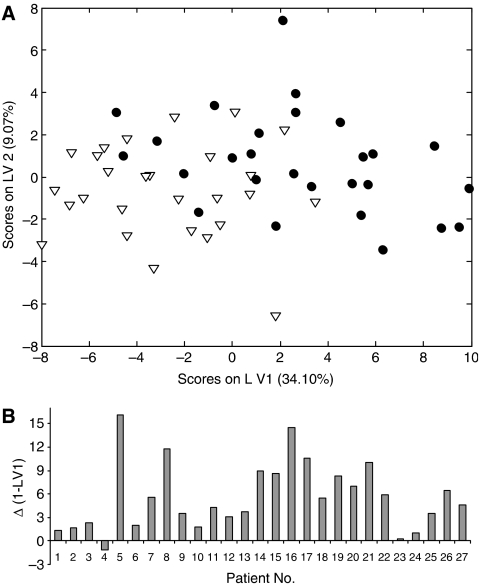
Partial least squares discriminate analysis (PLSDA) of serum proteomic features before/during HCC. (**A**) Plots the data using two latent variables (LV1 and LV2). Filled circles represent pre-HCC samples and hollow triangles during-HCC samples. (**B**) Shows the change in LV1 associated with the development of HCC in each patient. PLSDA was performed using PLS_Toolbox (Version 3.5, Eigenvector Research, Manson, WA, USA) running in Matlab (Version 7.1, The MathWorks, Natick, MA, USA).

The serum proteome profiles of 27 patients that developed HCC were measured before and after diagnosis of HCC. The mean time between paired samples was 45 months (range 8–104 months). The data were analysed using paired Wilcoxon tests and partial least squares discriminate analysis (PLSDA) which identifies discriminatory linear combinations of proteomic features referred to as latent variables (LVs). Figure 1A shows sample scores on LV1 and LV2 of PLSDA of the proteomic profiles of the pre-HCC and during-HCC samples – some separation of the two groups is evident. More importantly, Figure 1B shows that LV1 changes in the same direction in 26 out of 27 patients; that is there are consistent proteomic changes that occur in these patients as they develop HCC. Wilcoxon tests show that 40 proteomic features are significantly (*P*<0.05) associated with the occurrence of HCC, 19 of these features with *P*<0.001. The 10 most statistically significant HCC associated proteomic features are listed in Table 1.

### The effect of tumour radiofrequency ablation on proteomic profiles

**Figure 2:**
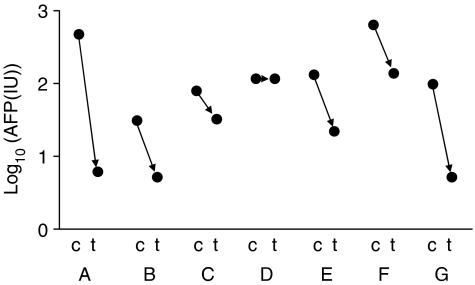
Serum AFP concentrations during HCC and post-treatment. Log_10_[AFP] is plotted for two time points (c=HCC, t=treated) for seven patients (labelled A–G).

Serum profiles were compared between paired samples from 18 HCC patients (10 common to the pre-HCC/HCC comparison) before and at least one month post tumour radiofrequency ablation (mean time between samples 15 months, range 1–57 months). Active tumour remnants were not detectable in the arterial phase of the CT scan in the samples from patients in the post-treatment phase indicating that complete local control of tumour was achieved in all cases. AFP was elevated in seven of the patients with HCC. As expected, in these, AFP substantially decreased following treatment, in six cases reaching either the reference range or the moderately elevated values frequently encountered in patients with active HCV cirrhosis (Figure 2). In the patient without a significant decrease in the serum AFP level, the level was moderately elevated and probably of extratumoural origin. Wilcoxon tests were used to identify significant differences in the SELDI peaks before and after treatment; eight of the 125 proteomic features change with *P*<0.05 between the pre- and post-treatment samples, however, none have *P*<0.001. The mean intensities of all eight of the peaks with *P*<0.05 continue the trend away from pre-HCC levels.

**Figure 3:**
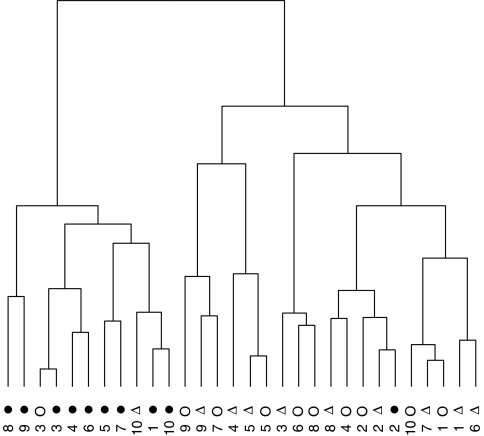
Cluster analysis of serum profiles from 10 patients pre-HCC, during-HCC and post-treatment. The intensities of 32 significant peaks were used to cluster the samples using the ‘dendrogram’ function in *R* (http://www.r-project.org). Each patient is numbered with the pre-HCC profile represented by a filled circle, the during-HCC profile with a hollow triangle and the post-treatment by a hollow circle.

For 10 patients sera were taken before HCC, with established HCC and post-treatment. All peaks with *P*<0.05 (Kruskal–Wallis test) between the before HCC, during-HCC and post-treatment were used to perform a cluster analysis (Figure 3). The pre-HCC samples cluster away from the during-HCC and post-treatment samples indicating that, in the majority of patients, the post-treatment serum profile is more similar to the during-HCC than the pre-HCC profile.

### Peak identification

**Figure 4:**
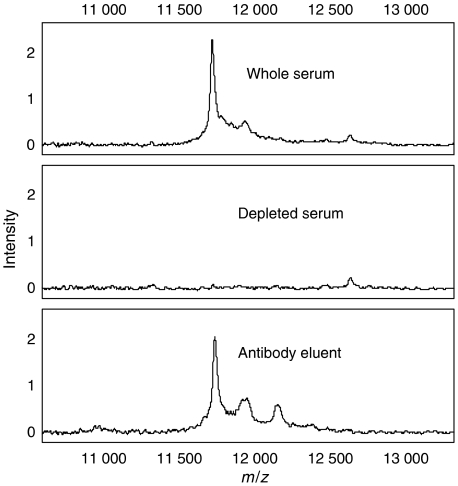
*β*2-Microglobulin immunoSELDI. IMAC spectra of whole serum (top), serum depleted with a *β*2-microglobulin antibody (middle) and the proteins captured by the antibody (bottom panel).

The proteomic feature at *m*/*z* 11720 that was highly significantly altered during HCC (average intensity increase of 80%) was selected for protein identification. The *m*/*z* 11720 peak was purified by anion exchange chromatography, RP-HPLC and SDS-PAGE. A band with ∼12 kDa mobility was excised from the gel and trypsinised. Three tryptic peptides from *β*2-microglobulin (sequence mass 11 715) were detected (S_20_NFLNCYVSGFHPSDIEVDLLK_41_, V_49_EHSDLSFSK_58_, V_82_NHVTLSQPK_91_) representing 42% sequence coverage. A peak at *m*/*z* 11925 co-purified with the *β*2-microglobulin and may correspond to a modified form of *β*2-microglobulin. Figure 4 shows the ‘immunoSELDI’ confirmation that the peak at *m*/*z* 11720 peak is indeed *β*2-microglobulin.

## DISCUSSION

AFP is currently in widespread use to monitor patients with chronic liver disease with a view to early detection of HCC development. However, concentrations are elevated in only about 30% of early HCC (Sherman, 2001). Therefore, there is a great need for more effective biomarkers for screening, early diagnosis and monitoring of treatment. Several studies have now suggested that SELDI profiling of serum can detect HCC in a background of chronic liver disease (Poon *et al*, 2003; Paradis *et al*, 2005; Schwegler *et al*, 2005; Ward *et al*, 2006b). These studies have usually involved comparison of serum from groups of patients with and without HCC, and very few ‘longitudinal’ studies, involving time-dependent proteomic changes in the same patient have been undertaken. In this study, we show that proteomic changes correlate with HCC development by comparing samples from patients before and at the time when the diagnosis of HCC was established. By comparing different stages of disease progression in the same group of patients we control for possible differences (other than those associated with HCC) introduced by inadvertent patient selection bias. Importantly, this approach can establish when the proteomic changes commence and if they are reversed by effective therapy. We find HCC associated proteomic changes that correlate with the onset of HCC but, unexpectedly, these changes do not return towards normal (pre-HCC) following radiofrequency ablation of the tumour.

We have identified one of the 13 proteomic features most strongly associated with HCC as *β*2-microglobulin. This is a particularly reassuring finding since *β*2-microglobulin has previously been shown (using immunoturbometric methods) to be markedly elevated in the serum of hepatitis C patients with HCV-related HCC when compared to HCV positive patients without HCC (36±16.5 *μ*g ml^−1^ *vs* 2.3±0.8 *μ*g ml^−1^; *P*<0.0001) or healthy subjects (1.6±0.4 *μ*g ml^−1^; *P*<0.0001) (Malaguarnera *et al*, 2000). However, it is unlikely that *β*2-microglobulin alone could act as a biomarker specifically for HCC as serum levels of *β*2-microglobulin are also elevated in patients with chronic inflammatory disease, viral disease (e.g. AIDS) and lymphomas (Cooper and Plesner, 1980). *β*2-Microglobulin might nonetheless add power to a multimarker HCC diagnostic test. Furthermore, and in keeping with our previous experience (Ward *et al*, 2006a, 2006b and 2006c), the identification of this peak as *β*2-microglobulin suggests that the profile is, at least in part, characterised by intact proteins rather than fragments of degraded proteins.

The lack of a ‘return to normal’ following apparently effective treatment (radiofrequency ablation) is, perhaps, surprising although there is precedent. Pusztai *et al* reported that resection of breast tumours did not produce a return to normal of SELDI profiles and work from our laboratory indicates that the same is true for lung cancer (Pusztai *et al*, 2004; Ward *et al*, 2006a). The half-lives of known tumour biomarkers in the serum are in the order of a few days and several tumour markers, including AFP are known to return towards normal shortly after successful treatment (Johnson and Williams, 1980). We found that AFP did indeed fall in six out of seven patients in this current study. This is not the case for the SELDI profiles and hence they would not appear to be useful for monitoring therapy, although a role in diagnosis is not precluded.

There are several possible explanations as to why the SELDI profiles still report ‘HCC’ following treatment. The first one would be that although tumour burden is greatly reduced there is still sufficient residual disease to maintain the HCC profile. This seems unlikely since follow-up did not show any short-term local or distant recurrence in our patients. It is also conceivable that the SELDI profile reports indirectly on the presence of the tumour by detecting the host response to the tumour that may persist after destruction. Another possibility is that SELDI detects changes in the liver that precede HCC. It is noteworthy that HCV patients with cirrhosis and HCC are at a very high risk of new primary HCC when they are not cured of their HCV infection (Yamanaka *et al*, 2005). Thus, the proteomic changes could be the hallmark of premalignant lesions that persist after the ablation of the first malignant tumour.

It seems likely that proteomic changes associated with HCC exist (in addition to AFP) which ‘return to normal’ following effective treatment. Although our current study failed to identify such markers it does identify a SELDI profile that is predictive of the presence or short-term occurrence of HCC at the moment of diagnosis. This fact is particularly interesting as most of the tumours were diagnosed at an early stage making them eligible for radiofrequency ablation. Various predictive factors of the occurrence of HCC have previously been reported and validated such as age, BMI and the severity of cirrhosis (Nair *et al*, 2002; Fattovich *et al*, 2004). Such factors, mainly epidemiological, are not susceptible to changes in the short-term. Therefore, it is likely that SELDI profile changes between prediagnosis and diagnosis samples reflect other phenomena that might have additional and independent predictive value. This hypothesis needs to be further explored by the close follow-up of patients with HCV cirrhosis who will be screened for HCC and repeated SELDI serum profiling.

## Figures and Tables

**Table 1:** The 10 serum proteomic features most significantly associated with the onset of HCC

***m*/*z* ratio of proteomic feature**	***P*- value**	**Mean change intensity (HCC/pre-HCC)**
11720	4.8 × 10^−5^	1.82
11925	5.5 × 10^−5^	1.76
2725	7.3 × 10^−5^	0.30
8925	7.3 × 10^−5^	1.25
8180	9.6 × 10^−5^	0.42
5900	0.00013	2.00
2640	0.00021	0.52
6105	0.00021	2.14
3965	0.00027	0.50
6120	0.00027	2.04

The table shows the mass to charge ratio of the peaks, the *P-*value (Wilcoxon test, *n*=27) and the mean peak intensity in the HCC sera relative to the pre-HCC sera.
